# Reconstruction of 12-Lead Electrocardiogram from a Three-Lead Patch-Type Device Using a LSTM Network

**DOI:** 10.3390/s20113278

**Published:** 2020-06-09

**Authors:** Jangjay Sohn, Seungman Yang, Joonnyong Lee, Yunseo Ku, Hee Chan Kim

**Affiliations:** 1 Interdisciplinary Program in Bioengineering, Seoul National University Graduate School, Seoul 03080, Korea; jjaysohn@melab.snu.ac.kr (J.S.); ysmgreen@melab.snu.ac.kr (S.Y.); 2 Mellowing Factory Co. Ltd., Seoul 03080, Korea; joonnyonglee@melab.snu.ac.kr; 3 Department of Biomedical Engineering, College of Medicine, Chungnam National University, Daejeon 34134, Korea; yunseo.ku@cnu.ac.kr; 4 Department of Biomedical Engineering, College of Medicine, Seoul National University, Seoul 03080, Korea; 5 Institute of Medical & Biological Engineering, Medical Research Center, Seoul National University, Seoul 03080, Korea

**Keywords:** LSTM network, patch-type device, reconstructed electrocardiogram, synthesized ECG, ubiquitous healthcare, hypertension, cardiovascular monitoring

## Abstract

Reconstructing a standard 12-lead electrocardiogram (ECG) from signals received from electrodes packed into a patch-type device is a challenging task in the field of medical instrumentation. All attempts to obtain a clinically valid 12-lead ECG using a patch-type device were not satisfactory. In this study, we designed the hardware for a three-lead patch-type ECG device and employed a long short-term memory (LSTM) network that can overcome the limitations of the linear regression algorithm used for ECG reconstruction. The LSTM network can overcome the issue of reduced horizontal components of the vector in the electric signal obtained from the patch-type device attached to the anterior chest. The reconstructed 12-lead ECG that uses the LSTM network was tested against a standard 12-lead ECG in 30 healthy subjects and ECGs of 30 patients with pathologic findings. The average correlation coefficient of the LSTM network was found to be 0.95. The ability of the reconstructed ECG to detect pathologic abnormalities was identical to that of the standard ECG. In conclusion, the reconstruction of a standard 12-lead ECG using a three-lead patch-type device is feasible, and such an ECG is an equivalent alternative to a standard 12-lead ECG.

## 1. Introduction

A 12-lead electrocardiogram (ECG) is a standard lead system used in clinical practice. A standard 12-lead ECG was designed to show the frontal components of a three-dimensional electric vector in six limb leads and the horizontal components in six precordial leads. In the frontal plane leads (I, II, III, aVR, aVL, aVF), the vectorial components of the frontal plane in I, II, and III are mathematically described as II = I + III. Augmented leads, aVR, aVL, and aVF, can also be derived from leads I, II, and III. Therefore, if two among leads I, II, and III can be obtained, we can reconstruct six frontal plane ECGs. Precordial lead (V1−V6) attachment sites are located anteriorly covering only half of the chest for the convenience of the patient. For the standard 12-lead ECG to be obtained, 10 electrodes are usually attached [1], however, attaching this large number of electrodes is not suitable for monitoring purposes.

Therefore, previous studies proposed limited lead systems for the reconstruction of the standard 12-lead ECG [2]. The most widely used limited lead system is the EASI lead system [3]. This system uses five electrodes instead of 10 electrodes. Even though the EASI system requires fewer electrodes, the electrodes still need to cover a large area of the upper chest. Not only the EASI lead system, but also many other special lead systems, were investigated in previous studies for the reconstruction of the 12-lead ECG. In addition to an effort to reduce the number of electrodes, several investigators [4,5] showed the feasibility of obtaining a 12-lead ECG from the signals obtained from a small area of the chest. In order to be used as the wearable device, not only should the number of electrodes be reduced, but the attachment sites should also be limited to only a small area.

The most common method to reconstruct the 12-lead ECG from a limited lead set is a linear regression (LR) because the electrical system of the human heart-torso is theoretically linear and quasi-stationary [6]. Reconstructing a 12-lead ECG, based on the linearity, has been widely used by many researchers, but there has been the opinion that the electrogenesis of the heart is not a purely linear process [7]. Therefore, the recommendation to improve performance was to use neural networks for the purpose of reconstruction [8], when noise and uncertainty caused by possible electrode misplacement are present [9].

In this study, we designed three lead patch-type devices with four electrodes inside each, and made an optimal model for the synthesis of a standard 12-lead ECG. Lead positions were determined based on the previous study [5]. We adopted the use of a recurrent neural network using a long short-term memory (LSTM) cell which is widely used in time series modeling [10]. Models are developed for each individual, that is, a personal set of equations are used. We made the reconstruction model with 10 s data. It was our intent to make the model with 10 s data because 10 s data is obtained in one go with a standard 12-lead ECG examination. Therefore, this personal model would not impose additional inconvenience on the user, other than obtaining a single standard 12-lead ECG. We validated the reconstructed ECG by comparing it with a standard 12-lead ECG in terms of the identicalness of parameters in normal subjects, and in terms of the detectability of pathologic findings in patients.

## 2. Materials and Methods

### 2.1. Subjects

We recruited 30 normal subjects and 30 patients. The 30 normal subjects (M:F 17:13, mean age: 42.2 ± 3.2) were negative for cardiac symptoms and showed normal echocardiograms. Additionally, 30 in-hospital patients (M:F 12:18, mean age: 53.1 ± 4.3), whose ECGs were recorded for their clinical needs, were enrolled. Informed consent was obtained for all normal subjects and patients. This study was approved by the institutional review board of Seoul National University Hospital (IRB No. 1707-094-870).

### 2.2. Experimental Setup

#### 2.2.1. Hardware

**Figure 1:**
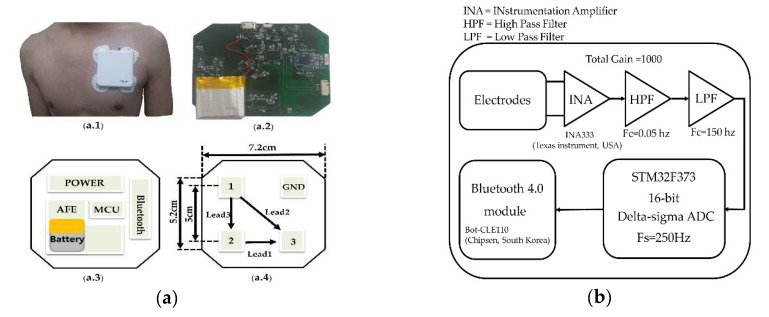
(**a.1**) Picture of the device attached on a patient. (**a.2**) Customized circuit. (**a.3**) Components of the front side. (**a.4**) Components of the back side. (**b**) Block diagram showing the signal processing and data acquisition of the device.

We established a system which was composed of a patch type device, an analysis program, MATLAB (Mathworks, MA, USA), and a commercial standard ECG device (MAC5500, GE, USA). A patch-type device was designed to measure a three-lead ECG from the left-upper chest, which is shown in Figure 1. The distance between each electrode’s location was 5 cm to get a reliable and strong ECG signal based on the signal-to-noise ratio reported in the previous studies [11,12]. A previous study reported that reconstructed ECGs from three leads obtained from the left-upper chest resulted in the highest correlation with the standard 12-lead ECG. An analog circuit was designed to prevent the distortion of the ECG baseline, which may have affected the displacement of the ST segment. The ECG signal was band-pass filter between 0.05–150Hz, according to recommendation by the American Heart Association [13] and amplified by a factor of 1000. Signals were digitized using a 16-bit sigma-delta analog-to-digital converter (STM32F373, STMicroelectronics, Switzerland) at 250 Hz and were transmitted by a Bluetooth module (Bot-CLE110, Chipsen, South Korea) to a data processing PC. A high pass filter frequency was 0.05 Hz, which was vulnerable to noise. Therefore, we obtained signals in the static situation to avoid the noise.

#### 2.2.2. Software

**Figure 2:**
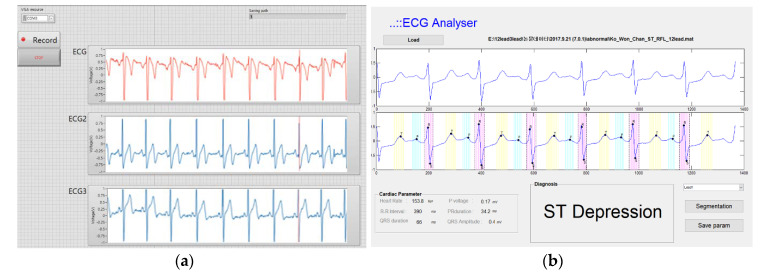
(**a**) Screenshot of three-lead ECG (electrocardiogram) data acquisition program. (**b**) Screenshot of ECG reconstruction and analyzing program using ECGPUWAVE.

The data acquisition program was made using Labview (National Instrument, Austin, TX, USA). It can receive the three-lead ECG signal continuously and save the file as a text file. As shown in Figure 2, the operator set the comport number and this was transmitted by a Bluetooth module (Bot-CLE110, Chipsen, South Korea) to a data processing PC. Waveforms subsequently transferred from the device were plotted on the graph. To compare the signal quality, we extracted a diagnostic parameter, such as P amplitude, PR interval, QRS voltage and duration, and QT duration. We programed an analysis program using MATLAB that can align an ECG signal and segment P, Q, R, S, and T waves. First, we detected the R peak using the Pan and Tompkins algorithm [14]. This algorithm uses the characteristics of the QRS complex which predominantly contains 8–16 Hz components, the slope of which changes rapidly. We can detect the R peak easily by a band-pass filter and derivatives. After obtaining the R peak in this way, other peaks, such as P, Q, S, T, are detected using ECGPUWAVE [15] which is the PhysioNet’s open source algorithm. After getting the onset, offset, and peak of P, Q, R, S, and T we calculated the parameters, such as the amplitude of each peak and interval, which is shown in Figure 2. In regards to the pathologic ECG findings in this study, we looked for the presence of a pathologic Q wave, ST depression, ST elevation, T wave inversion, and wide QRS complex. ECG diagnosis was made based on the following criteria. Wide QRS: QRS duration ≥ 120 ms, ST elevation: elevation of ST segment ≥ 0.1 mV 0.08 ms after J point, ST depression: depression of ST segment ≥ 0.1 mV 0.08 ms after J point, pathologic Q wave; amplitude ≥ 25% of R wave and ≥ 0.04 ms in duration. With all the clinical results, double-checking procedures were done by the clinician.

### 2.3. Measurement Protocols (Data Acquisition Program)

**Figure 3:**
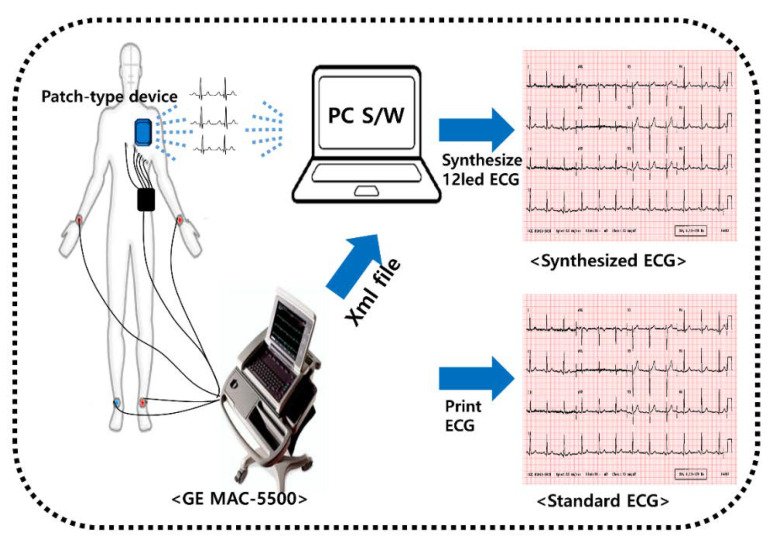
System for the clinical validation of a reconstructed ECG from a patch-type device. ECG: electrocardiogram.

Patient preparation using our patch-type device is shown in Figure 3. When the standard 12-lead ECGs were obtained for 10 s using a commercial ECG device (MAC 5500, General Electric, USA) with a sampling rate of 250 Hz, data from the three-lead patch-type device were also recorded. Ag/AgCl electrodes were used. Signals were obtained in the supine position and subjects were requested not to move at the time of signal acquisition. The data from the ECG device were transferred wirelessly to a PC using a Bluetooth module. Signals from our patch system were digitally stored for the synthesis of a 12-lead ECG using linear and nonlinear methods. The synthesized 12-lead ECG was compared with the standard 12-lead data obtained by the ECG machine. All ECG diagnoses were confirmed by the cardiologist.

### 2.4. Reconstruction Model

**Figure 4:**
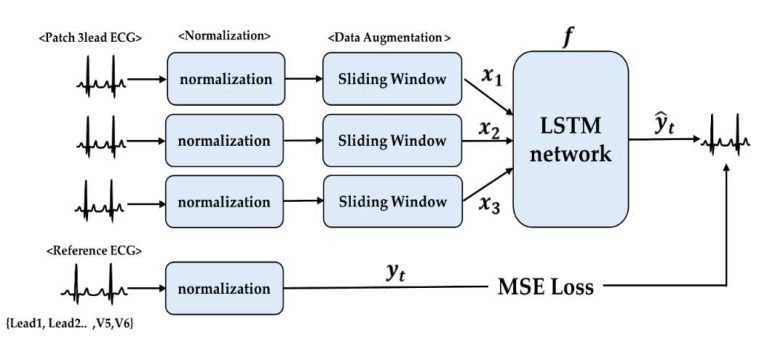
Flow of the proposed algorithm (x1: lead1 ECG data, x2: lead2 ECG data, x3: lead3 ECG data, MSE: mean square error). ECG: electrocardiogram.

We developed the models based on two major assumptions of the electrical vectors of the human heart. One is LR and the other is the LSTM network. As mentioned in the introduction, each model was established with 10 s data. The size of the training data was determined by 5 s data (half of the saved data). To verify the algorithm, the transformation matrix and LSTM network were determined by 5 s training data and tested by another 5 s of data. For the training of the LSTM network, we performed a 1 s sliding window with a 4 ms time step, as shown in Figure 4.

As the training and test datasets were obtained consecutively in our experiment, we validated the comparable outcome when these two datasets were obtained 5 days apart for five normal volunteers in an identical test setting. 

#### 2.4.1. Linear Regression

To reconstruct 12-lead ECGs from the three-lead ECGs using LR, we employed a method widely used in the previous studies [4,16,17]:

Y = α∗X+β(1)

Y contains the target standard 12-lead ECG which we want to reconstruct. α is the transformation matrix which is needed to reconstruct the ECG. X is a combination of the reduced number of ECG data. β is a matrix which contains vectors of error. The matrix for which we want to get α is obtained using the least squares method. Transformation matrix α is obtained by using the 5 s training data taken from a total of 10 s of data. Once α and β were obtained, we synthesized a 12-lead ECG with the test data. Transformation matrix X is obtained individually. Since the subjects have their own electric vector, the electrical activity of the heart can be assumed to be a single fixed location dipole.

#### 2.4.2. Long Short-Term (LSTM) Network

**Figure 5:**
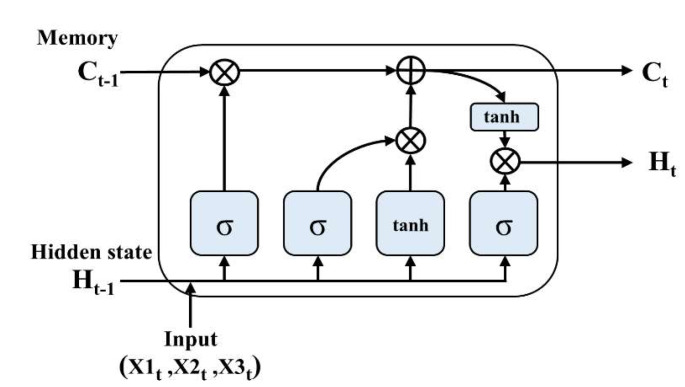
Structure of employed LSTM (X1: lead1 ECG data, X2: lead2 ECG data, X3: lead3 ECG data). LSTM: long short-term memory.

The LSTM cell, which was designed by Sepp Hochreiter and Jürgen Schmidhuber [18], is used for time series modeling. The LSTM network is efficient for ECG modeling because these cells solve the “gradient vanishes or explodes” issue by incorporating gate units and memory cells. Important features of data can be efficiently delivered and maintained in the LSTM during calculation, which can be achieved through an input gate, forget gate, and output gate [18]. The LSTM structure is shown in Figure 5. The input signals are a component of the three-lead ECG from the device, and the final output is a reconstructed ECG.

The weights of input, forget, and output gates are represented as $w_{i}$,$\;w_{f}$, and $w_{o}$ respectively. Each part of LSTM can be represented as:

(2) $$I_{t}=\sigma \left(w_{i}x_{t}+w_{i}h_{t-1}\right)$$

(3) $$F_{t}=\sigma \left(w_{f}x_{t}+w_{f}h_{t-1}\right)$$

(4) $$\tilde{C_{t}}=\tan h\left(w_{c}x_{t}+w_{c}h_{t-1}\right)$$

(5) $$O_{t}=\sigma \left(w_{0}x_{t}+w_{0}h_{t-1}\right)$$

In the above equations, σ denotes the sigmoid activation. The input gate annotated as vector $I_{t}$ is calculated by the hidden state $h_{t-1}$ from the former LSTM cell and input vector $x_{t}$ of the current stage. In the forget gate, vector $F_{t}$ is determined by the hidden state $h_{t-1}$ and the input $x_{t}$ determines whether the state vector c from time t−1 is carried or not, as shown in (4):

(6) $$C_{t}=\sigma \left(C_{t-1}F_{t}+\tilde{C_{t}}I_{t}\right)$$

(7) $$H_{t}=O_{t}\tan h\left(C_{t}\right)$$

As shown in (6), the input gate $I_{t}$ regulates the calculation $\tilde{C_{t}}$ in $C_{t}$. The output $H_{t}$ is generated by applying a tanh activation function on $C_{t}$ which is used to regulate the $O_{t}$, which is the output gate. In this study, we made 12 LSTM networks which perform the process of reconstructing the output ${\hat{y}}_{t}$ by $x_{1},\;x_{2},\;x_{3}$, which means each lead of data can be represented as:

(8) $${\hat{y}}_{t}=f\left(W_{1}x_{1}+W_{2}x_{2}+W_{1}x_{3}+b\right)$$

For network training, the reconstruction error should be minimized, known as cost function J:

(9) $$J=\frac{1}{m}\overset{m}{\underset{i=1}{\sum }}h\left(y_{t},{\hat{y}}_{t}\right)$$

$y_{t}$ is the standard ECG (lead1~V6) and m is the number of signals. $h\left(y_{t},{\hat{y}}_{t}\right)$ is the reconstruction error, which is represented by the mean square error between the original signal $y_{t}$ and its reconstructed signal ${\hat{y}}_{t}$, as shown in Figure 4. It can be expressed as:

(10) $$h\left(y_{t},{\hat{y}}_{t}\right)=||y_{t}-{\hat{y}}_{t}||{}^{2}$$

In this study, the number of layers and neurons were three and 50, respectively, which was decided experimentally by comparing the performances of various numbers of networks with training data. A batch size of 50 and an epoch number of 500 were used. The time-step was 250, so the input data size was 3 × 250 and the output data size was 1 × 250. We selected the Adam optimizer with a learning rate of 0.001. The performance of the network was estimated with test data that were not used to train the network.

**Figure 6:**
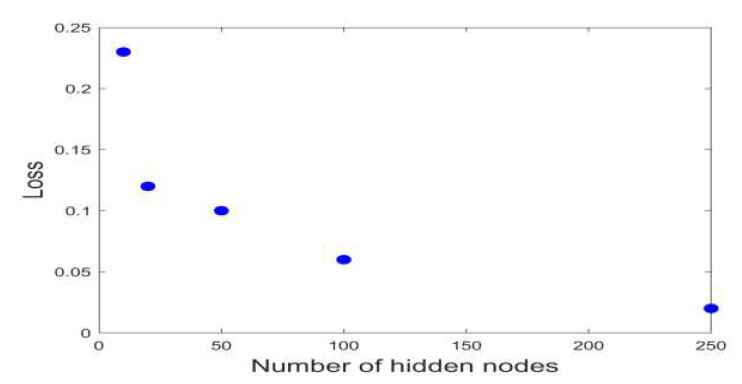
The loss values of reconstruction models based on the numbers of hidden nodes.

Training loss can be reduced by the increase in the hidden nodes. (Figure 6) However, we adopted a hidden node number of 50 to reduce the time that was consumed in training and to avoid overfitting.

### 2.5. Statistics

Standard 12-lead and reconstructed ECGs were compared using correlation and root mean square error. In the diagnosis of pathologic findings using reconstructed ECGs, sensitivity and specificity were obtained. In the comparison of numerical parameters in three groups, a repeated measures ANOVA was used with a significance level of *p* < 0.05.

## 3. Results

**Figure 7:**
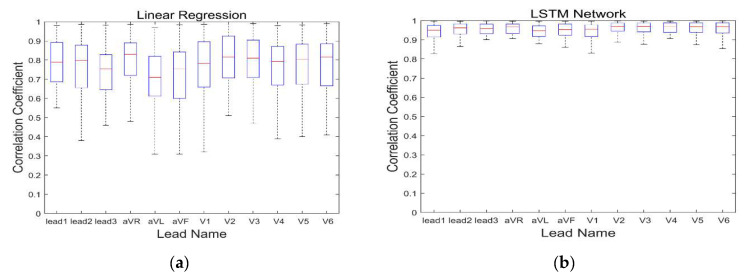
Correlation coefficients between standard 12-lead ECGs and synthesized ECGs. (**a**) Linear regression. (**b**) LSTM network. LSTM: long short-term memory

Correlation coefficients and root mean square errors (RMSE) between standard 12-lead and synthesized ECGs are shown in Table 1 and Figure 7.

### 3.1. Identicalness

We compared waveforms for identicalness using the root mean square error (RMSE) and correlation coefficient (CC). The CCs in LR and LSTM were 0.74 and 0.95, respectively. The RMSE in LR and LSTM were 53.16 μV ± 15.52 and 20.41 μV ± 8.45, respectively. Reconstructed ECGs using both methods showed considerable identicalness with the standard ECG, especially in ECGs reconstructed by the LSTM network. However, clinical usefulness does not solely lie in identicalness. If pertinent information regarding the magnitude and direction of the electric vector can be delivered to the physician, clinical usefulness can still be maintained. 

### 3.2. Measurement of Normal Parameters

Both LR and LSTM methods did not show significant differences in axis, PR interval, QRS duration, QT duration, or T wave amplitudes from standard 12-lead ECGs, as shown in Table 2. The reconstructed ECG using LR showed statistically significant lower voltages in QRS and P wave amplitude. The reconstructed ECG using LSTM also showed a tendency for lower voltage in QRS and P wave amplitudes but the difference was not statistically significant. 

### 3.3. Detection of Pathologic Findings

Multiple pathologic findings can be seen in a single patient, and in this study 66 total pathologic findings were noted in 30 patients. Sensitivity and specificity were calculated from these 66 pathological findings. Diagnostic sensitivity and the specificity of reconstructed ECGs in predicting various pathologic ECG findings are shown in Table 3.

#### 3.3.1. Left Ventricular Hypertrophy (LVH)

**Figure 8:**
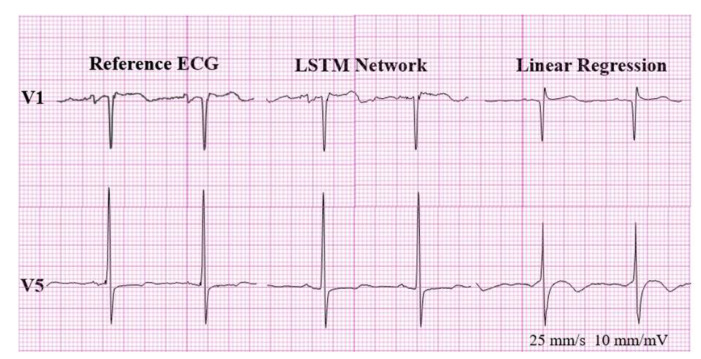
False negative result for LVH.

Although specific, the sensitivity of detecting LVH was quite poor in the reconstructed ECG using LR. This finding is expected considering the finding that the reconstructed ECG using LR showed significantly lower voltage when compared to the normal subjects. Thus, it did not fulfil the diagnostic criteria of LVH (S wave voltage in lead V1 + R wave voltage in lead V5 or V6 ≥ 35 mV).(Figure 8)

#### 3.3.2. ST Change

**Figure 9:**
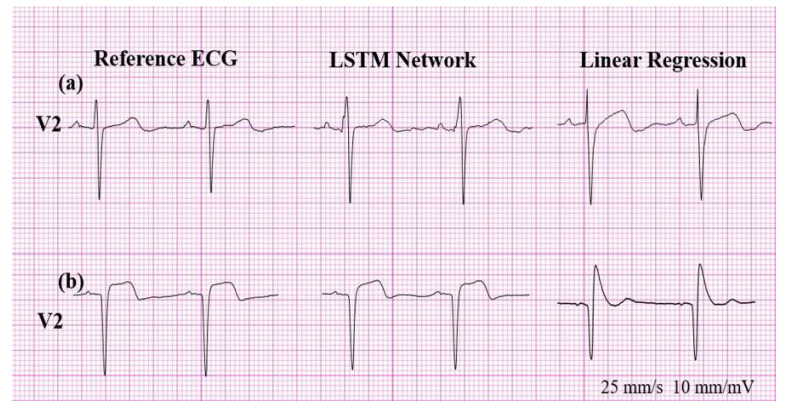
(**a**) False positive for ST elevation and (**b**) false negative result for ST depression.

In contrast to predicting the presence of Q wave or T wave inversion, sensitivities in detecting ST segment changes in the reconstructed ECG using LR were quite poor. Therefore, the reconstructed ECG using LR is clinically not applicable in the detection of ST changes, both in ST depression and ST elevation. However, reconstructed ECGs using LSTM are free of this limitation, as shown in Figure 9.

#### 3.3.3. Wide QRS

**Figure 10:**
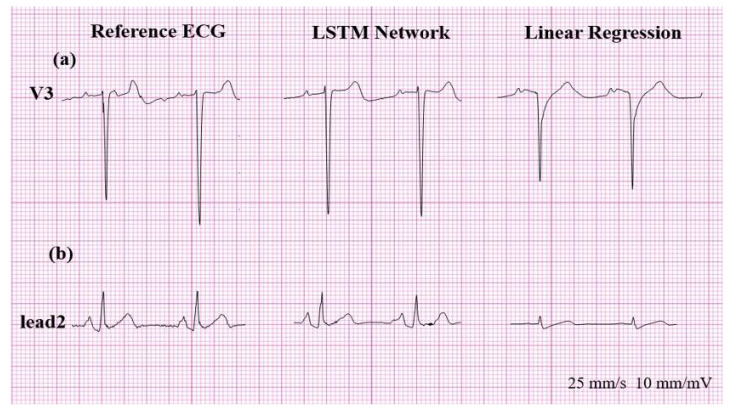
(**a**) False positive wide QRS in the reconstructed ECG using LR. (**b**) Large differences in voltage between limb and precordial leads were usually seen in patients who showed the false positive wide QRS in the reconstructed ECG using LR. LR: linear regression, ECG: electrocardiogram

The reconstructed ECG can detect, with high sensitivity, wide QRS (QRS duration ≥ 120 ms). However, in four patients, the ECG reconstructed using LR showed false positive wide QRS, as shown in Figure 10.

Owing to this false positive result, the specificity of detecting wide QRS was found to be only 83% in the ECG reconstructed by LR. In addition, in three patients with false positive wide QRS, the ECG showed reduced voltages in the limb leads. We hypothesized that the ECG reconstructed by LR is vulnerable to this type of error, as our leads system predominantly reflects frontal plane vectors and has a limitation in detecting the horizontal components of the QRS vector.

### 3.4. Training and Test Performed with a Time Interval

For five normal volunteers, training was carried out 5 days in advance of the test. The mean CCs and RMSEs of the standard 12-lead and synthesized ECGs using LR were 0.73 ± 0.21 and 53.41 ± 16.42 μV, respectively. Those using the LSTM network were 0.95 ± 0.04 and 17.64 ± 7.22 μV, respectively.

## 4. Discussion

The reconstructed ECG with a reduced number of leads has been studied for a long time due to the clinical need for continuous monitoring in the intensive care unit with diagnostic test machines or for application in wearable devices. These studies aimed to reduce the number of electrodes from the standard lead positions [19,20,21]. The three or four lead positions selected in these studies were leads I, II, V3, and V5 or V6. For the best performance, precordial leads that can represent the vector component in the horizontal plane (V3, V5 or V6) were included. In one such study [21], when only one precordial electrode location was used in the reconstruction, the leads used in capturing the horizontal component of the vector were reduced, and the CC dropped to 0.854 (0.642–0.912). Our patch system also has an intrinsic limitation in capturing the horizontal component of this vector.

The spread out locations required for the lead attachment sites are a critical limitation for their usage in wearable devices. For this purpose, the numbers of electrodes need to be reduced, the leads need to be comfortable to wear [22], and the lead locations should be close enough to be packed into a patch-type system [23,24]. This patch system has the additional advantage of easy adaptation to Bluetooth and the incorporation of an additional sensor if required.

In our study, the reconstruction of ECG using LSTM showed a similar performance compared to the previous studies (Table 4), the majority of which were achieved by the linear regression method. However, our study aimed at the feasibility of reconstruction using signals obtained by the patch device. The lead system incorporated in the small-sized patch used in our study is limited in terms of the spatial resolution of the electric vector of the ECG. Even if we had to reconstruct ECG in a personalized manner, this drawback is less problematic for application in a wearable device than are the widely separated multiple leads.

In the measurement of normal parameters, there were no significant differences between the standard 12-lead ECG and the ECGs reconstructed by LR and by the LSTM network, although the reconstructed ECG using LR showed a slightly reduced QRS and P amplitude. Even if the reconstructed ECG by LR tends to show a reduced voltage in QRS, these ECGs are well suited to be used in the majority of situations. However, our study showed that in our system, the ECG reconstructed using LR is not practically useful in detecting ST changes, which is one of the important reasons for monitoring the ECG in patients with ischemic heart disease. In our study, this limitation can be overcome by reconstructing an ECG using the LSTM network. In detecting the presence of pathological Q wave and T wave inversion, the ECG reconstructed using LR has practically no limitations. Therefore, we assume that an ECG reconstructed using LR is good at detecting the changes in the direction of the electric vector, but insensitive in the detection of the magnitude of changes for this vector, such as the ST segment elevation and depression. In addition to this insensitivity, in ECGs reconstructed using LR, there were several cases of false positive results in ST changes, which is another problematic issue when applied to the purpose of monitoring patients with ischemic heart disease.

The most conspicuous false positive result for the ECG reconstructed using LR was the false positive wide QRS. This is because a wide QRS morphology can possibly be mistaken for a serious ventricular arrhythmia, unless an initial check for the possibility of a false positive wide QRS is conducted. We cannot precisely explain the cause of this phenomenon but believe that this phenomenon was more likely to be seen in patients with a large discrepancy in amplitudes between the limb and precordial leads. The proposed LSTM network, however, was not likely to result in this false positive wide QRS.

**Figure 11:**
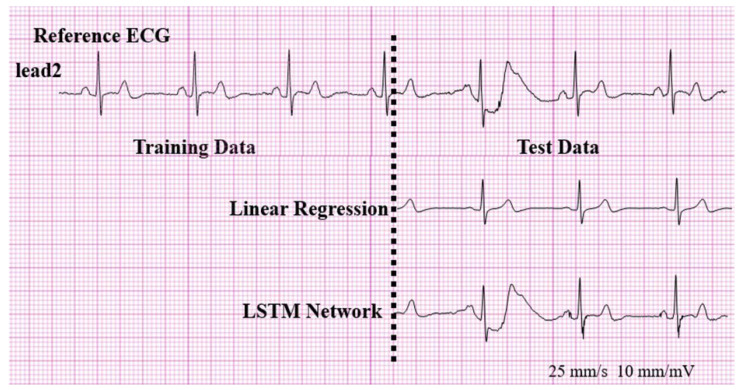
Reproduction of the ventricular ectopic beats that are not present in the 5 s training data.

There might be a criticism that an ECG reconstructed using the LSTM network in our study was simply repeating the training data. However, this criticism can be refuted by the fact that the ECG reconstructed using the LSTM network in our study can accurately reproduce the ventricular ectopic beats with quite a different QRS morphology, as shown in Figure 9, that is not present in the 5 s training data. This finding suggests that the LSTM network is not simply repeating the training data, rather it is finding a nonlinearity between the patch-type ECG and a standard ECG. Previous studies used artificial neural networks (ANNs) in the reconstruction of ECGs [8]. These methods showed good performances, but when we applied an ANN to our system, it could not reconstruct the ectopic beat, as shown in Figure 11. In our study, an recurrent neural network(RNN) using LSTM cells was used, as we assumed that the LSTM cell, which has the advantage of reflecting time information, is better suited for the reconstruction of ECGs.

For practical applications, we limited the data to 5 s for training purposes; these data can be obtained in a single ECG examination. Nevertheless, the LSTM network requires an average of 30 min to 1 h for the training of an individual subject. Therefore, the LSTM network cannot be applied immediately after the signal acquisition but requires a preparation period. In addition, as our study was performed in the static position, the applicability of our method in a dynamic condition should be validated in future studies. In conclusion, the reconstruction of a 12-lead ECG using an LSTM network from a three-lead patch-type device is feasible and valid for the measurement of parameters and in the detection of pathological findings in patients.

## Figures and Tables

**Table 1:** Mean correlation coefficients and RMSE between standard 12-lead and synthesized ECGs.

Outcome		I	II	III	aVR	aVL	aVF	V1	V2	V3	V4	V5	V6
Correlation Coefficient (mean)	Linear Regression	0.76	0.75	0.70	0.77	0.68	0.71	0.75	0.77	0.78	0.75	0.74	0.75
	LSTM Network	0.92	0.96	0.95	0.96	0.93	0.95	0.93	0.96	0.96	0.96	0.96	0.95
Root Mean Square Error (μV) (mean)	Linear Regression	23.40	74.46	64.09	45.16	68.26	32.05	34.69	48.58	58.90	59.49	66.53	62.27
	LSTM Network	20.81	17.42	18.01	47.76	20.85	18.02	20.68	15.56	16.12	16.06	16.09	17.58

RMSE: root mean square error, ECG: electrocardiogram.

**Table 2:** CC and RMSE between standard 12-lead and reconstructed ECGs.

ECG Parameter		Standard ECG	Synthesized ECG by Linear Regression	Synthesized ECG by LSTM Network	*p*-value
Axis (degree)		58.6 ± 35.2	60.2 ± 35.0	61.0 ± 35.0	n.s
P wave: Amplitude in lead II (mV)		1.73 ± 0.63 *	1.17 ± 0.93 *	1.57 ± 0.72	0.004
PR interval (ms)		152.7 ± 22.6	143.4 ± 35.1	148.5 ± 27.8	n.s
QRS	Duration (ms)	57.4 ± 11.0	56.9 ± 9.2	56.0 ± 10.3	n.s
	Total Voltage (mV)	140.7 ± 31.2 *	110.2 ± 30.6 *	139.9 ± 32.4	0.004
QT duration (ms)		57.4 ± 11.0	56.9 ± 9.2	56.0 ± 10.3	n.s
T wave amplitude (mV)	V4	2.5 ± 2.1	2.6 ± 1.7	2.6 ± 2.3	n.s
	V5	2.5 ± 2.0	2.6 ± 1.9	2.5 ± 2.2	n.s
	V6	2.1 ± 1.6	2.1 ± 1.5	2.2 ± 1.7	n.s

*: *p* < 0.05, CC: correlation coefficient, RMSE: root mean square error, ECG: electrocardiogram.

**Table 3:** Sensitivity and specificity of pathologic findings in the reconstructed ECGs.

	Synthesized ECG by Linear Regression		Synthesized ECG by LSTM Network	
	Sensitivity (%)	Specificity (%)	Sensitivity (%)	Specificity (%)
LVH (*n* = 11)	64	100	100	100
ST elevation ant. and Inf. combined (*n* = 6)	63	95	100	100
ST depression in inf. and lat. combined (*n* = 13)	46	88	100	100
Wide QRS (*n* = 6)	100	83	100	100
Pathologic Q (*n* = 8)	86	96	100	100
T wave inversion in V4-6 (*n* = 12)	83	100	92	100

ECG: electrocardiogram.

**Table 4:** Previous studies on reconstruction ECGs.

Study	Source ECG	Synthesis Method	Subjects	Performance (Average CC)
Atoui, H et al. [8]	Subset of standard ECG (I, II, V2)	Neural Network and Linear Regression	120 patients	0.948
Trobec, R et al. [16]	Three bipolar lead	Linear Regression	30 normal, 35 patients	0.959 (median)
Lee, D et al. [25]	Three bipolar lead	Artificial Neural Network	14 normal	0.920
Zhang, Q et al. [26]	Subset of standard ECG (I, II, V2)	Linear Regression, LSTM network	20 patients	0.820
Tomašić, I et al. [27]	Three bipolar lead	Regression Trees	20 normal, 22 patients	0.985
Zhy et al. [28]	Subset of standard ECG (I, II, V2)	Linear Regression	39 patients	0.947
Our system	Three bipolar lead	LSTM network	30 normal, 30 patients	0.950

ECG: electrocardiogram.

## References

[1] Plonsey J.M. (1995). 12-lead ECG system. Bioelectromagnetism.

[2] Finlay D.D., Nugent C.D., Kellett J.G., Donnelly M.P., McCullagh P.J., Black N.D. (2007). Synthesising the 12-lead electrocardiogram: Trends and challenges. Eur. J. Intern. Med..

[3] Dower G.E., Yakush A., Nazzal S.B., Jutzy R.V., Ruiz C.E. (1988). Deriving the 12-lead electrocardiogram from four (EASI) electrodes. J. Electrocardiol.

[4] Tomasic I., Frljak S., Trobec R. (2013). Estimating the Universal Positions of Wireless Body Electrodes for Measuring Cardiac Electrical Activity. IEEE. Trans. Biomed. Eng..

[5] Lee H.J., Lee D.S., Kwon H.B., Kim D.Y., Park K.S. (2017). Reconstruction of 12-lead ECG Using a Single-patch Device. Methods Inf. Med..

[6] Hadzievski L., Bojovic B., Vukcevic V., Belicev P., Pavlovic S., Vasilijevic-Pokrajcic Z. (2004). A novel mobile transtelephonic system with synthesized 12-lead ECG. IEEE Trans. Inf. Technol. Biomed..

[7] Gulrajani R.M. (1998). The forward and inverse problems of electrocardiography. IEEE Eng. Med. Biol. Mag..

[8] Atoui H., Fayn J., Rubel P. (2004). A neural network approach for patient-specific 12-lead ECG synthesis in patient monitoring environments. Comput. Cardiol..

[9] Atoui H., Fayn J., Rubel P. (2010). A novel neural-network model for deriving standard 12-lead ECGs from serial three-lead ECGs: Application to self-care. IEEE Trans. Inf. Technol. Biomed.

[10] Hou B., Yang J., Wang P., Yan R. (2019). LSTM Based Auto-Encoder Model for ECG Arrhythmias Classification. IEEE Trans. Instrum. Meas..

[11] Puurtinen M., Hyttinen J., Malmivuo J. (2005). Optimizing bipolar electrode location for wireless ECG measurement-analysis of ECG signal strength and deviation between individuals. Int. J. Bioelectro. magn..

[12] Puurtinen M., Viik J., Hyttinen J. (2009). Best electrode locations for a small bipolar ECG device: Signal strength analysis of clinical data. Ann. Biomed. Eng..

[13] Bailey J.J., Berson A.S., GarsonJr A., Horan L.G., Macfarlane P.W., Mortara D.W., Zywietz C.Z. (1990). Recommendations for standardization and specifications in automated electrocardiography: Bandwidth and digital signal processing. A report for health professionals by an ad hoc writing group of the Committee on Electrocardiography and Cardiac Electrophysiology of the Council on Clinical Cardiology, American Heart Association. Circulation.

[14] Pan J., Tompkins W.J. (1985). A real-time QRS detection algorithm. IEEE Trans. Biomed. Eng..

[15] Laguna P., Jane R., Caminal P. (1994). Automatic detection of wave boundaries in multilead ECG signals: Validation with the CSE database. Comput. Biomed. Res..

[16] Trobec R., Tomasic I. (2011). Synthesis of the 12-lead electrocardiogram from differential leads. IEEE Trans. Inf. Technol. Biomed..

[17] Tomasic I., Trobec R. (2014). Electrocardiographic systems with reduced numbers of leads-synthesis of the 12-lead ECG. IEEE Rev. Biomed. Eng..

[18] Hochreiter S., Schmidhuber J. (1997). Long short-term memory. Neural Comput..

[19] Scherer J.A., Jenkins J.M., Nicklas J.M. (1989). Synthesis of the 12-lead electrocardiogram from a 3-lead subset using patient-specific transformation vectors. An algorithmic approach to computerized signal synthesis. J. Electrocardiol.

[20] Cady L.D. (1969). Computed relationship of standard electrocardiographic leads. Med. Res. Eng..

[21] Nelwan S.P., Kors J.A., Meij S.H., Van Bemmel J.H., Simoons M.L. (2004). Reconstruction of the 12-lead electrocardiogram from reduced lead sets. J. Electrocardiol.

[22] Gao Y., Soman V.V., Lombardi J.P., Rajbhandari P.P., Dhakal T.P., Wilson W., Poliks M., Ghose K., Turner J.N., Jin Z. (2019). Heart Monitor Using Flexible Capacitive ECG Electrodes. IEEE Trans. Instrum. Meas..

[23] Lobodzinski S.S. (2013). ECG patch monitors for assessment of cardiac rhythm abnormalities. Prog. Cardiovasc. Dis..

[24] Torfs T., Smeets C.P., Geng D., Berset T., Auwera J.V., Vandervoort P., Grieten L. (2014). Clinical validation of a low-power and wearable ECG patch for long term full-disclosure monitoring. J. Electrocardiol.

[25] Lee D., Kwon H., Lee H., Seo C., Park K. (2020). Optimal Lead Position in Patch-Type Monitoring Sensors for Reconstructing 12-Lead ECG Signals with Universal Transformation Coefficient. Sensors.

[26] Zhang Q., Frick K. All-ECG: A Least-number of Leads ECG Monitor for Standard 12-lead ECG Tracking during Motion. Proceedings of the 2019 IEEE Healthcare Innovations and Point of Care Technologies.

[27] Tomasic I., Trobec R., Lindén M. (2015). Can the regression trees be used to model relation between ECG leads. International Internet of Things Summit.

[28] Zhu H., Pan Y., Cheng K.T., Huan R. (2018). A lightweight piecewise linear synthesis method for standard 12-lead ECG signals based on adaptive region segmentation. PLoS ONE.

